# Requirements for Durability Improvement of Conductive Patterns Permeated in Textiles under Cyclic Tensile Deformation

**DOI:** 10.3390/mi10110721

**Published:** 2019-10-25

**Authors:** Tomoya Koshi, Ken-ichi Nomura, Manabu Yoshida

**Affiliations:** Sensing System Research Center (SSRC), National Institute of Advanced Industrial Science and Technology (AIST), 1-1-1 Higashi, Tsukuba, Ibaraki 305-8565, Japanyoshida-manabu@aist.go.jp (M.Y.)

**Keywords:** flexible electronics, E-textiles, printing process, conductive pattern, permeation

## Abstract

Conductive patterns on textiles are one of the key components for electronic textiles (E-textiles). The patterns with deeper permeation of inks into the textiles show better durability against cyclic tensile deformation. However, other requirements for improving the durability and the behavior of resistance under deformation are still unclear. In this study, the resistance during cyclic tensile deformation was measured with changing conditions, and the resistance variation was analyzed while considering the stress variation. Silver inks were printed on a plain weave, and the pattern width and tensile direction against weft yarns were changed. Measurements confirmed that the resistance increased less with wider pattern widths and when the tensile direction was horizontal to the axis of the weft yarns. Through scanning electron microscopy (SEM) observation, we also confirmed that the growth rate of cracks, at the crossing point of yarns, was changed by the tensile direction. These results indicate that the durability is improved when the electricity path redundancy within the pattern is robust, and the crack growth rate at the yarn crossing points is low. The analysis also confirmed both increasing and decreasing behavior of resistance during stretching in the cyclic tensile deformation, indicating the behavior results from the stress variation of a plain weave.

## 1. Introduction

In recent years, flexible electronic devices, such as displays [1,2], batteries [3,4], and sensor array sheets [5,6], have been developed by many research groups [7,8,9,10,11]. In particular, flexible electronic devices embedded in textiles, known as E-textiles, have attracted attention for developing new fashions [12], human-machine interfaces [13,14], biological information monitoring systems [15,16,17], and so on [18,19,20,21,22,23,24,25]. In previous studies, weaving/knitting functional yarns within textiles [26,27,28,29] and printing functional inks on textiles [30,31,32] were used to fabricate electric circuits on textiles. Compared with weaving/knitting conductive yarns, the processes of printing conductive ink are more promising for manufacturing E-textiles. This is because typical printing processes, such as screen printing, are compatible with established manufacturing processes of electronics.

For E-textiles manufactured using printing processes, printed conductive patterns are a key component. Conductive patterns printed on textiles require both high conductivity and durability against bending or stretching. Generally, conductive patterns gradually lose their conductivity by cyclic deformation until conduction failure. Previous studies reported that conductive patterns with deeper permeation of the ink into textiles have better durability against cyclic tensile deformation [33,34]. These studies indicate that the fracture process of the conductive pattern by tensile deformation is significantly different between the pattern with less and deep permeation. When the permeation is not deep, the fracture process must be similar to that of patterns on conventional substrates, such as plastic/rubber films, because the structure is almost a double layer structure of inks and textiles. In this case, the crack propagation and peeling of ink must occur all over the pattern, and finally, conduction failure is caused by the crack and peeling. Therefore, solutions proposed in previous studies are useful for durability improvements, such as using stretchable inks and improving adhesion between the patterns and textiles. On the other hand, when the permeation is deep, the fracture process must be different from that with less permeation, because the structure is almost a textile-like structure. This means that requirements for durability improvement are also different. However, there are few studies focusing on the deeper permeation of inks specifically for the durability improvement, and therefore, other requirements and the fracture process, that is, the behavior of resistance variation under the cyclic tensile deformation, are still not clear.

In this study, we assumed the following factors must have an important role in the durability improvement: (1) redundancy of the electricity path in the patterns, and (2) crack growth rate at the crossing point of yarns. When conductive ink is printed on a textile, the ink permeates and coats each yarn. Each yarn obtains conductivity by the ink coating and permeation. The crossing points of yarns are electrically and mechanically connected through the ink only. In this way, the yarns form redundant networks of electrical paths in the conductive pattern. The crack initiation and propagation must occur at the crossing points of yarns because of stress concentration, where the conductive patterns are deformed by tensile deformation. When the conductive patterns are composed of too few conductive yarns, that is, when the redundancy of electrical paths is not robust, the conductive patterns easily lose their conductivity because the electrical paths are interrupted by cracks. This effect is worsened with a higher crack growth rate in the conductive pattern.

Therefore, in this study, to examine the effect of redundancy and crack growth rate on the durability of conductive patterns permeated into textiles, the electrical resistance of patterns under cyclic tensile deformation was measured. Scanning electron microscopy (SEM) was also used to observe cracks within the textile. In addition, the variation in resistance with respect to stress variation was analyzed to deepen our understanding of this behavior.

## 2. Materials and Methods

**Figure 1:**
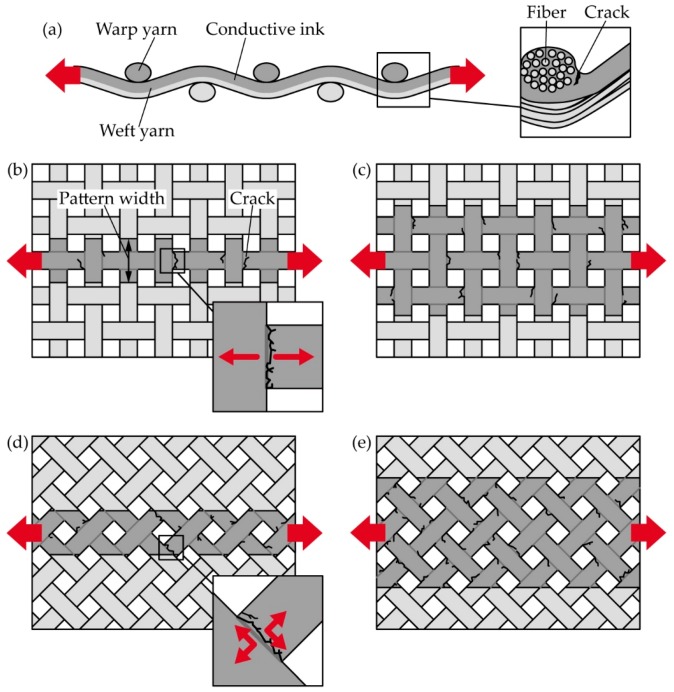
Schematic illustrations of a deformed plain weave with a permeated conductive ink: (**a**) cross-section. Top side schematics with horizontal weave (**b**,**c**) and diagonal weave (**d**,**e**).

In this study, a plain weave was used as a textile. The plain weave has a simple crisscross pattern of warp and weft yarns. Figure 1a–e show schematic illustrations of a deformed plain weave with permeated conductive ink. The ink permeates and coats each yarn, and cracks occur at yarn crossing points, as shown in Figure 1a. To change the redundancy of electrical paths, the pattern widths were varied. For example, comparing Figure 1b and c, more conductive yarns are included for a wider pattern width (Figure 1c). In this case, the redundancy must be more robust than the case with less conductive yarns, given that complete interruption of the electrical path, by cracks, is less likely. To change the crack growth rate at yarn crossing points, the tensile direction against the plain weave was changed, such that we used a horizontal and diagonal weave. For the horizontal weave (Figure 1b,c), only weft yarns are stretched along the axial direction of the weft yarns. In this case, the opening force occurs at the crossing point, as shown in Figure 1b. The crack initiations occur around the crossing points and propagate perpendicularly to the tensile force direction, due to the stress concentration. While for the diagonal weave (Figure 1d,e), both warp and weft yarns are deformed by tensile deformation. In this case, the opening and tearing forces occur at the crossing point, as shown in Figure 1d. The initial cracks propagate diagonally to the tensile force direction. These different forces, at the crossing points, change the crack growth rate. A conventional conductive ink, plain weave, and printing process were used: silver ink, plain weave cotton, and screen printing, respectively. In this study, we used an epoxy-based silver ink. The epoxy-based silver ink has better conductivity and availability, although it costs a little more than other functional inks. Therefore, epoxy-based silver ink is one of the most used functional inks in the electronics industry. Whereas, plain weave cotton and screen printing dominated their respective industries some time ago.

**Figure A1:**
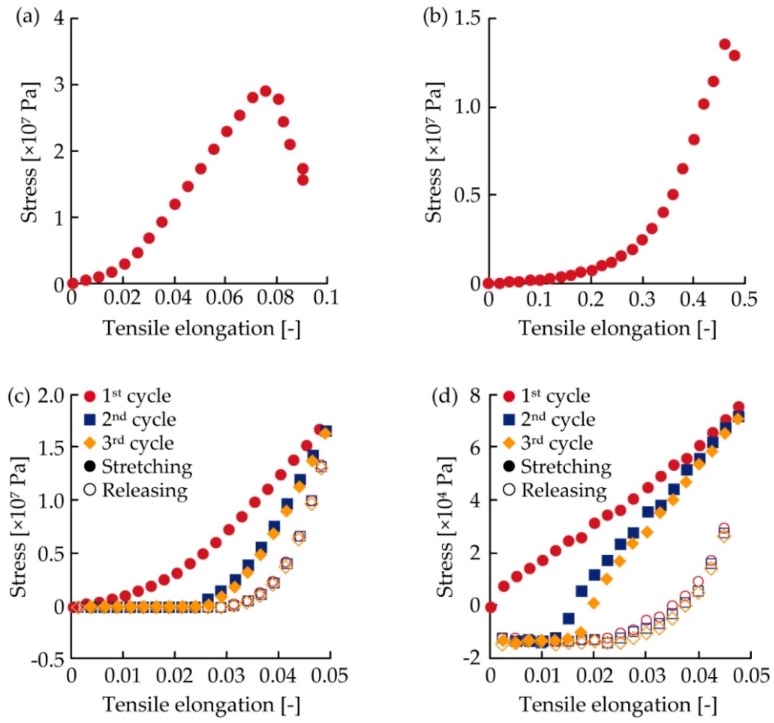
Mechanical property of a plain weave cotton under tensile deformation. (**a**) Horizontal and (**b**) diagonal weave under single tensile deformation. (**c**) Horizontal and (**d**) diagonal weave under tensile deformation of 3 cycles.

Samples used in the experiments were prepared as follows: silver ink (MP501SO/6322E, Mino Group Co., Ltd., Gifu, Japan) was printed onto a commercially available plain weave cotton, by screen printing. The silver ink mainly consists of silver flakes, epoxy resin, and glycol-ether-type organic solvent. The median diameter of the flake is 1.93 μm, and the viscosity is ~5.3 Pa·s at a shear rate of 100 s^−1^. The diameter and pitch of yarns of plain weave were 0.2 mm and 0.3 mm, respectively. We measured the mechanical property of plain weave cotton, and it is shown in Figure A1. The shape of the pattern was straight. The conductive patterns on the screen mask had a length of 20 mm, and the widths were 0.1 mm, 0.5 mm, and 1.0 mm. The actual measurement value of widths varied because of the silver ink permeation into the yarns. Note, this is described in detail later. The contact pads were also patterned on both sides of the conductive patterns. This was done to obtain an electrical connection with the measuring equipment. The samples were baked at 80 ℃ for 30 min, in an oven, to obtain the conductivity of the silver ink. To achieve electrical connection with the measuring equipment, conductive threads were bonded to contact pads using conductive adhesive ink.

**Figure 2:**
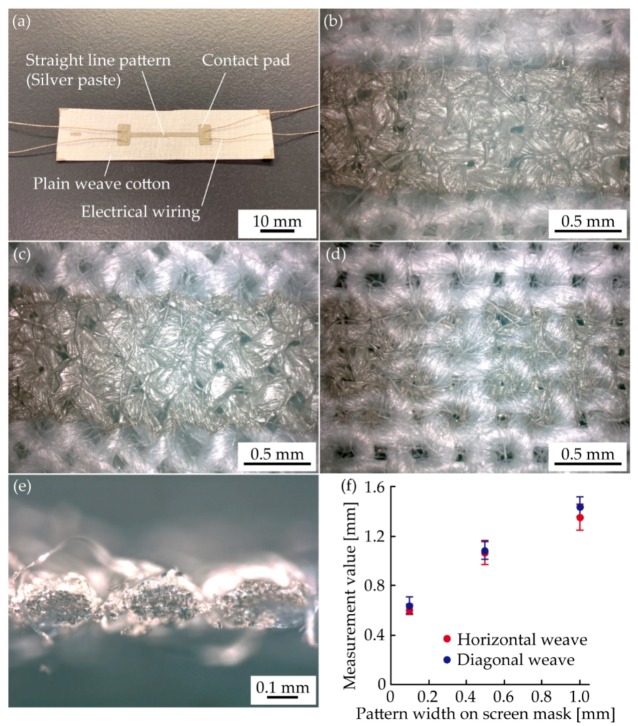
(**a**) Optical image of the prepared sample. Enlarged optical images of a conductive pattern with (**b**) horizontal and (**c**) diagonal weaves. (**d**) Bottom side and (**e**) cross-sectional optical images of a conductive pattern with the horizontal weave. (**f**) Relationship between pattern width on a screen mask and actual measurement value of pattern width.

**Figure 3:**
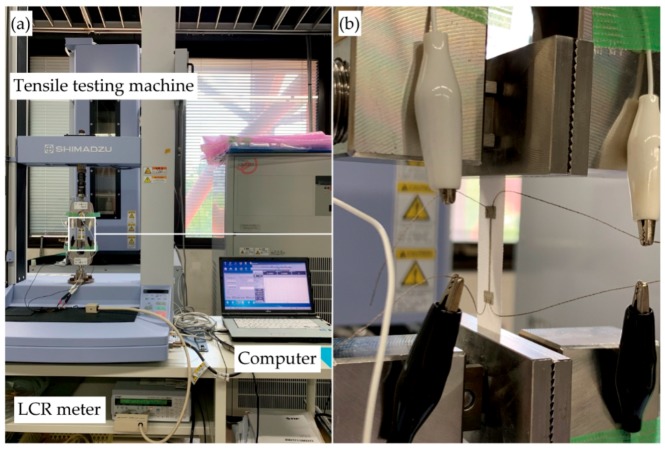
(**a**,**b**) Setup of resistance measurement under cyclic tensile deformation.

Figure 2a shows an optical image of the prepared sample. Figure 2b,c show the enlarged top side optical images of conductive patterns permeated into the horizontal and diagonal weaves, respectively. These optical images show that the printed silver ink permeated into the yarns in both cases. Figure 2d shows the bottom side of the conductive pattern with the horizontal weave. Its cross-section image is shown in Figure 2e. We also confirmed that the silver inks permeated into each yarn. Figure 3f shows the relationship between the pattern width on the screen mask and the actual measurement value for the horizontal and diagonal weaves. The actual measurement values for the horizontal weave were 0.6, 1.1, 1.4 mm, respectively. These were 0.4–0.5 mm wider than those on the screen mask. The same was true for the diagonal weave samples. This result shows that the effect of permeation was essentially the same regardless of the type of weave. However, note that permeation will differ for different materials and printing conditions.

Figure 3a,b show a setup of resistance measurement under cyclic tensile deformation. A prepared sample was clamped in a tensile testing machine (AGS-X, Shimadzu Co., Kyoto, Japan), and the sample was electrically connected to an LCR meter (ZM2371, NF Co., Kanagawa, Japan). The DC resistance of the conductive pattern was measured using a four-probe method. The applied voltage to the sample was 1.4 V. In this study, the resistance change rate was defined as (*R*-*R*_0_)/*R*_0_, where *R* and *R*_0_ are resistance and initial resistance of conductive pattern, respectively. The resistance change rate was used to compare the resistance variation with changing conditions. Three samples were used for each condition. The tensile testing machine was elongation driven. The rate of tensile deformation was approximately 7 mm/min. When the plain weave is stretched to a certain deformation amount, compressive stress perpendicular to the tensile direction occurs between the weft and warp yean at the crossing point. We consider that the resistance must show unique behavior by the perpendicular compressive stress, and therefore a tensile elongation of 0.05 was applied to the sample to analyze the behavior. Tensile elongation was calculated as the ratio of the clamp displacement to the initial distance between the clamps. Regarding the number of tensile cycles, some previous studies conducted experiments with 1000 or more cycles to guarantee durability for wearable applications. However, our objective is to examine the effect of redundancy and crack growth rate on the resistance change behavior; therefore, tensile deformation cycled 100 times from the viewpoint of the understandability of the measured data. The LCR meter and tensile testing machine were connected to a computer, and the measured resistance and tensile force were recorded. For the SEM observations, the samples were observed with a VHX-D510 (Keyence Co., Osaka, Japan).

## 3. Results and Discussion

### 3.1. Resistance Measurement with Changing Condition

**Figure 4:**
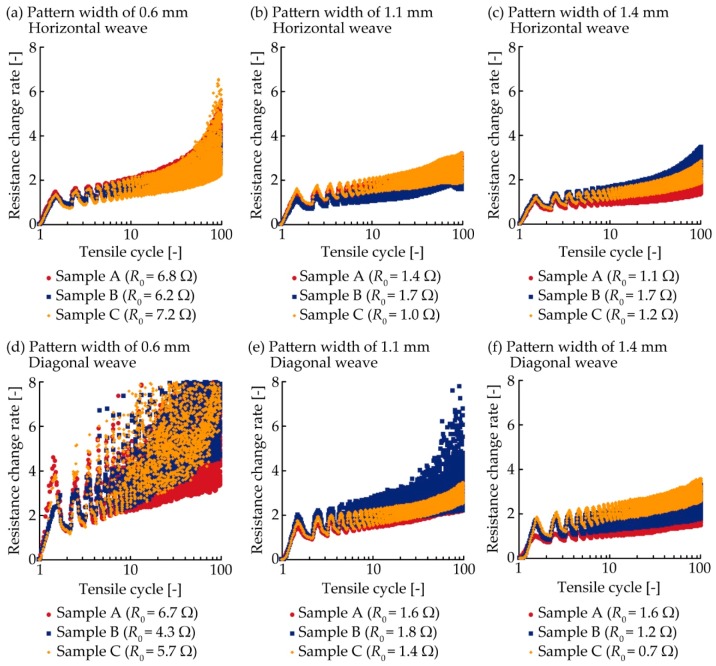
Resistance variation of conductive patterns printed on a plain weave under cyclic tensile deformation of 5%. (**a–c**) Pattern widths of 0.6, 1.1, and 1.4 mm with horizontal weave. (**d–f**) Pattern widths of 0.6, 1.1, and 1.4 mm with diagonal weave.

Figure 4 shows the resistance variation determined by the number of tensile deformation cycles. For the 0.6 mm pattern width and horizontal weave (Figure 4a), the resistance change rate of each sample increased from 0 to approximately 1.5 and then decreased to approximately 0.9 during the 1^st^ cycle of tensile deformation. This variation is caused by stretching and releasing, respectively, during a tensile deformation cycle. After the 1^st^ cycle, the resistance repeated this variation. The resistance change rate variation range expanded and shifted to greater values as the number of tensile cycles increased, varying from approximately 2.3 to 6.1 during the 100^th^ cycle. For the 1.1 mm pattern width and horizontal weave (Figure 4b), although the resistance increased, as with Figure 4a, the resistance values around the 100^th^ cycle were lower than that in Figure 4a. The resistance change rate around the 100^th^ cycle also had lower values than that in Figure 4a for the 1.4 mm pattern width and horizontal weave (Figure 4c). This confirms that the conductive patterns with a wider width have higher durability than those with narrower widths. For the 0.6 mm pattern width and diagonal weave (Figure 4d), the resistance change rate of all samples shows a significantly sharper variation during the 1^st^ cycle than that of any other samples or conditions. After the 1^st^ cycle, both the variation range and value increased more than the other conditions, as the number of tensile deformation cycles increased. For the 1.1 mm pattern width and diagonal weave (Figure 4e), one of the samples showed a sharp increase after approximately 30 cycles. However, the other samples showed lower resistance increases than the one shown in Figure 4d. For the 1.4 mm pattern width and diagonal weave (Figure 4f), all samples showed a lower resistance increase than that of the 0.6 mm pattern width. This also confirms that the durability of conductive patterns increases as the pattern width increases. Therefore, these results indicate that the durability of conductive patterns is improved when the electrical path redundancy within the pattern is robust. Additionally, the samples with horizontal weave tend to show lower resistance variation than that of diagonal weaves. This confirms that the conductive patterns with horizontal weaves have higher durability than those with diagonal weave.

**Figure 5:**
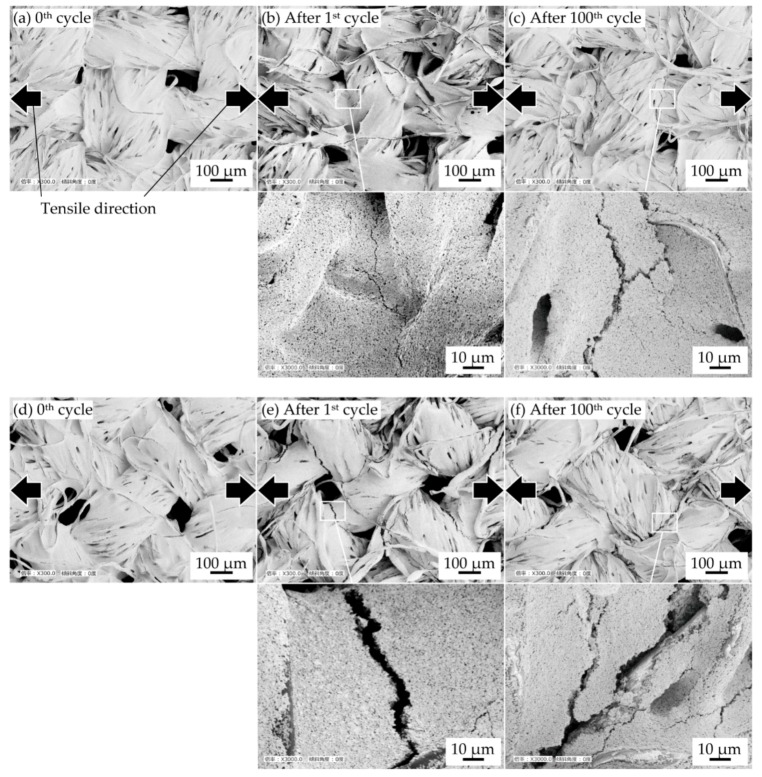
SEM images of conductive patterns printed on a plain weave before and after cyclic tensile deformation. Pattern width of 1.4 mm with horizontal weave (**a**) before stretching, after (**b**) the 1^st^ cycle and (**c**) 100^th^ cycle. Pattern width of 1.4 mm with diagonal weave (**d**) before stretching, after (**e**) the 1^st^ cycle and (**f**) 100^th^ cycle.

Figure 5 shows the SEM images of a sample with 1.4 mm-wide conductive patterns, before and after the cyclic tensile deformation. The conductive patterns with horizontal and diagonal weave are shown in Figure 5a––f, respectively. For the horizontal weave, the cracks do not occur before cyclic tensile deformation (Figure 5a). After the 1^st^ and 100^th^ cycle (Figure 5b,c), some cracks occurred at the crossing point of the warp and weft yarns. The direction of crack propagation is almost perpendicular to the tensile direction. Comparing samples after 1^st^ and 100^th^ cycles (Figure 5b,c), the number of cracks and crack openings after the 100^th^ cycle is greater than those after one cycle. This indicates that the increase in resistance is caused by crack initiation and propagation around the crossing points of warp and weft yarns. For the diagonal weave, the cracks also do not occur before the tensile deformation (Figure 5d) and occur at the yarn crossing points after the 1st cycle. The direction of crack propagation is almost diagonal to the tensile deformation. Compared to the samples with horizontal weaves after the 1^st^ cycle, more cracks occur, and the crack openings are wider in the samples with diagonal weave. This confirms that the crack growth rate was changed by the tensile direction against the yarns and that the cracks propagate more easily in the diagonal weave samples. Therefore, these results indicate that the conductive pattern durability is improved when the crack growth rate, at the crossing point, is lower. Regarding the direction effect of tensile deformation, Figure 5 indicates that, when the conductive pattern formed on horizontal weave is deformed vertically to the length direction, the cracks propagate parallelly to the length direction. In this case, the electrical paths in the pattern should be less interrupted by the crack. When the pattern on diagonal weave is deformed vertically to the length direction, the cracks propagate diagonally to both the deforming and length direction. In this case, the electrical paths should be interrupted as with the pattern deformed parallelly to the length direction.

### 3.2. Analysis of Resistance Variation Considering the Stress Variation

**Figure 6:**
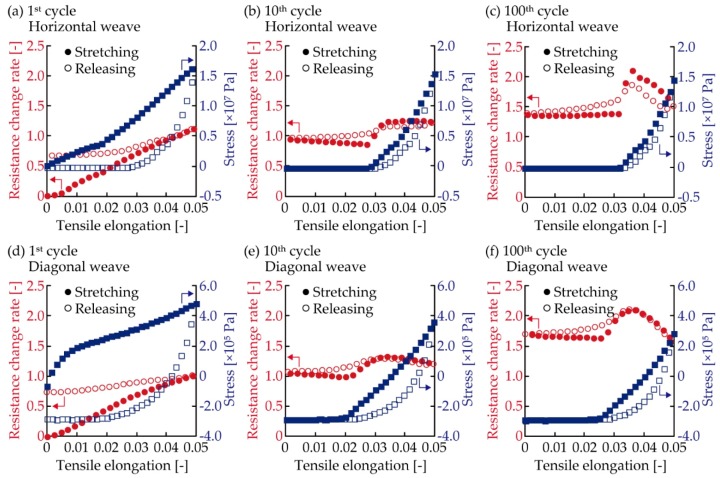
Resistance and stress variation for one of the samples with 1.4 mm-wide conductive patterns. Horizontal weave during (**a**) 1^st^, (**b**) 10^th^, and (**c**) 100^th^ cycle. Diagonal weave during (**d**) 1^st^, (**e**) 10^th^, and (**f**) 100^th^ cycle.

Figure 6 shows the relationship between the resistance and stress variation regarding one of the samples with 1.4 mm-wide conductive patterns. The resistance and stress variation during the 1^st^, 10^th^, and 100^th^ cycles are shown. The stress was obtained by dividing the measured tensile force by the initial cross-sectional area of the samples. During the 1^st^ cycle with the horizontal weave (Figure 6a), the resistance change rate increased in an almost linear manner; from 0 to 1.1 during the stretching, as the tensile elongation increased from 0 to 0.05. When the tensile elongation decreased from 0.05 to 0, the resistance change rate gradually decreased to 0.67. In Figure 6a, the stress increased from 0 to 17 MPa during the stretching, as the tensile elongation increased. When the tensile elongation decreased from 0.05, the stress decreased more sharply than it did during stretching. During the 1^st^ cycle with the diagonal weave (Figure 6d), the resistance and stress varied in a similar fashion to that shown in Figure 6a. The stress in Figure 6d is smaller than that shown in Figure 6a and shows a negative value during the releasing action. The negative value indicates compressive stress. During the 10^th^ cycle with the horizontal weave (Figure 6b), the resistance change rate slightly decreased as the tensile elongation increased from 0 to 0.03 during the stretching. Then, the resistance change rate sharply increased as the tensile elongation increased from 0.027 to 0.034, before slightly decreasing again after the tensile elongation increased from 0.034. During the releasing, the resistance repeated this slight variation. The stress in Figure 6b increased once tensile elongation reached 0.027 during the stretching, and also showed hysteresis in the stretching and releasing cycle. During the 10^th^ cycle with the diagonal weave (Figure 6e), the resistance and stress showed almost the same behavior as that shown in Figure 6b. During the 100^th^ cycle with horizontal and diagonal weaves (Figure 6c,f, respectively), the resistance increased and decreased more sharply than that shown in Figure 6b,e, but the behavior is similar. In Figure 6b,c,e,f, the resistance started to increase sharply when the stress began increasing during the stretching, and the resistance started to decrease when the stress changed from the negative to positive. These results show clear resistance variation during stretching in the cyclic tensile deformation, and the behavior corresponds to the stress variation. This indicates that the increasing and decreasing behavior of resistance results from the stress variation of the plain weave pattern.

**Figure 7:**
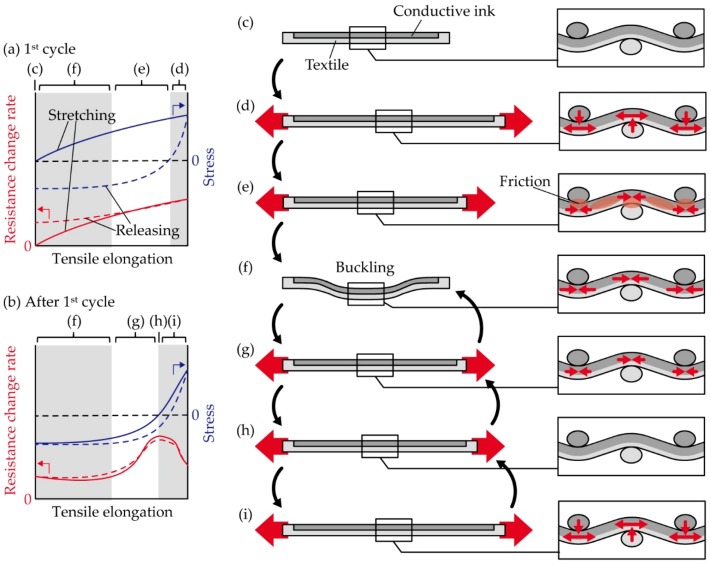
Schematic illustrations of a deformed sample under cyclic tensile deformation. Simplified resistance and stress variation (**a**) during the first cycle and (**b**) after the first cycle. (**c–i**) Schematic illustration of sample and stress caused in the sample.

Figure 7 shows the schematic illustrations of a deformed sample and the stress variation during the cyclic tensile deformation. We consider the mechanism of the observed behavior as follows. When the sample is stretched from the initial states (Figure 7c), tensile strain occurs in the samples, and the resistance increases (Figure 7d). When the tensile deformation is released, friction must occur in the yarn fibers and the contact surface at the crossing points of the warp and weft yarns, and therefore, the stress decreased more sharply than it did during stretching, and compressive stress was started in the sample. This hysteresis is known as a unique mechanical property of plain weave [35], and this behavior is also observed in Figure A1. When the tensile elongation decreases further, buckling starts to occur (Figure 7f), and therefore, the compressive stress converges to some value. During the decrease of tensile deformation, the resistance decreases because of the increasing compressive stress. Note that the resistance variation is also caused by the residual deformation of silver ink permeated into the yarns, and the crack initiation at the yarn crossing points. After the 1st cycle, when the elongation rate increases from 0, the buckling starts to release (Figure 7f). While the buckling releases, the compressive stress remains largely unchanged. When the buckling is entirely released, the compressive stress starts to decrease, and therefore, the resistance starts to increase (Figure 7g). When the stress changes from negative to positive, no stress occurs in the sample (Figure 7h). When the tensile stress starts to occur again, tensile stress occurs in the sample once again, but compressive stress perpendicular to the tensile stress direction also occurs between the weft and warp yean at the crossing point (Figure 7i). Then, the crack opening is decreased by the compressive stress, and therefore, the resistance decreases after increasing. When the tensile deformation is released, these deformations occur in reverse order.

## 4. Conclusions

Regarding the conductive patterns permeated into textiles, we clarified the requirements for improving the durability and resistance variation behavior under cyclic tensile deformation. To understand the requirements and the resistance variation behavior, the resistance of conductive patterns under cyclic tensile deformation was measured with changing conditions, and the resistance variation was analyzed while considering the stress variation. We considered that the electrical path redundancy within the conductive pattern and the crack growth rate at the yarn crossing points must play an important role in the textile durability. Therefore, the following parameters were changed in the experiments; the pattern width was changed to 0.6–1.4 μm and the tensile direction against the weft yarn was changed to horizontal and diagonal. The measurement confirmed that the resistance increased less when the pattern width was 1.4 mm and that the tensile direction was horizontal. The SEM observations also confirmed that the crack growth rate in the horizontal weave was lower than that of the diagonal weave. These results indicate that durability is improved when the electrical path redundancy is robust and the crack growth rate is low. The analysis confirmed both increasing and decreasing behavior during stretching in the tensile deformation cycle, indicating that the behavior results from the stress variation of the plain weave.

## Appendix A

The relationship between stress and tensile elongation of plain weave cotton under single/cyclic tensile deformation was measured, as shown in Figure A1. A tensile testing machine was used. The tensile direction against the plain weave was changed to horizontal and diagonal. Regarding the single deformation, the samples were stretched until broken. Regarding the cyclic deformation, the tensile elongation was 0.05, and the deformation of 3 cycles was applied to the samples.
